# Prevalence and risk factors of obesity among undergraduate student population in Ghana: an evaluation study of body composition indices

**DOI:** 10.1186/s12889-023-17175-5

**Published:** 2024-03-21

**Authors:** Christian Obirikorang, Evans Asamoah Adu, Enoch Odame Anto, Anthony Afum-Adjei Awuah, Angela Nana Bosowah Fynn, George Osei-Somuah, Patience Nyarkoa Ansong, Alexander Owusu Boakye, Ivy Ofori-Boadu, Yaa Obirikorang, Austin Gideon Adobasom-Anane, Eric NY Nyarko, Lois Balmer

**Affiliations:** 1 Department of Molecular Medicine, School of Medical Science, Kwame Nkrumah University of Science and Technology (KNUST), Kumasi, Ghana; 2 Kumasi Centre for Collaborative Research, Kumasi, Ghana; 3 Department of Medical Diagnostics, Faculty of Allied Health Sciences, Kwame Nkrumah University of Science and Technology, Kumasi, Ghana; 4 Centre for Precision Health, School of Medical and Health Sciences, Edith Cowan University, Western Australia, Australia; 5 Department of Nursing, Faculty of Health Sciences, Garden City University College, Kumasi, Ghana; 6 Department of Chemical Pathology, University of Ghana Medical School, University of Ghana, Accra, Ghana

**Keywords:** Adiposity, Obesity, Body composition, Undergraduate students, Risk factors

## Abstract

**Background:**

Obesity is a classified risk factor for several of the world’s leading causes of death. In this study, we combined information contained in body mass index (BMI), total percentage body fat (TPBF) and relative fat mass (RFM) to estimate obesity prevalence and examine the risk factors associated with obesity.

**Methods:**

The study recruited 1027 undergraduate students aged between 16 and 25 years using a cross-sectional study design and two-stage stratified random sampling between January and April 2019 from the Kwame Nkrumah University of Science and Technology, Kumasi, Ghana. Demographic, lifestyle, and family history of chronic disease data, were collected using a structured questionnaire. Bioelectrical impedance, along with height, weight, age, and gender, were used to estimate BMI and TPBF. The RFM was calculated using a published equation. The TPBF and RFM ranges were evaluated based on standard BMI thresholds and an informative combined obesity prevalence estimated in a Bayesian framework. Multiple logistic regression analysis was used to evaluate potential risk factors of overweight/obesity.

**Results:**

Concordance between BMI, TPBF and RFM for obesity classification was 84% among female and 82.9% among male students. The Bayesian analysis revealed a combined prevalence means of obesity of 9.4% (95%CI: 6.9-12.2%) among female students and 6.7% (95%CI:4.3-9.5%) among male students. The odds of obesity were increased between 1.8 and 2.5 for females depending on the classification index. A significant increasing trend of obesity was observed with university-level. A family history of obesity was associated with a high estimate of general, central, and high TPBF.

**Conclusion:**

Using multiple adiposity indicators conjointly in a Bayesian framework offers a greater power to examine obesity prevalence. We have applied this and reported high obesity prevalence, especially among female students. University level and family history of obesity were key determinants for obesity among the student population.

**Supplementary Information:**

The online version contains supplementary material available at 10.1186/s12889-023-17175-5.

## Introduction

Obesity is a classified risk factor for several of the world’s leading causes of death including cardiovascular diseases, diabetes, and various types of cancers [1]. It represents the 5th and 6th major level two public health problem among women and men, respectively, leading to the toll of death and disability worldwide [2]. Obesity stands out among the top leading causes of attributable disability-adjusted life years (DALYs) this is due to the rate of exposure increasing by more than 0.5% per year [3]. The prevalence of obesity has increased in pandemic dimensions over the past 50 years [4] with 650 million adults, 340 million adolescents and 39 million children classified as obese [5]. As the obesity pandemic continues, estimates indicate that approximately 167 million adults and children will become less healthy due to being overweight or obese by 2025 [4]. Especially in developing countries, the possible implications of obesity on current and future population health and healthcare spending are likely to be enormous [6].

According to existing nationwide data, the prevalence of being overweight and obese is estimated at 25.4% and 17.1%, respectively [7]. Among the adult Ghanaian population, obesity is higher in women than men and mimics the level of urbanization [7]. A meta-analysis involving 29,160 Ghanaian children (≤ 19 years) across sixteen studies reported 8.6% obesity and 10.7% overweight [8]. There exists a significant number of studies that quantify the burden of obesity in Ghana with a special focus on the general adult population [9–14], women [15–21], school-aged children [22–24], adolescents [25–27], healthcare workers [28–32], civil servants [33–38] and commercial workers [39]. However, knowledge and data about the experiences of being overweight and obese among young Ghanaian adults are inadequate. Among the few existing studies in Ghana [20, 40–42], there is an inconclusive estimate of those who are overweight/obese (4.2-39.3%) among the young adult population. This is due to population non-representativeness, that is, varying lifestyle habits and health-related behaviours of these age groups.

Among undergraduate students, which mainly represent the young adult population group, poor lifestyle habits, including decreased quality of diet and physical activity, sedentary lifestyle, alcohol use and smoking, as well as decreased quality sleep, are associated with obesity [42–44]. Also, the concurrence of altered eating behaviours (emotional eating, uncontrolled eating, and restrained eating), depression and poor sleep are estimated to be high among undergraduate students, mainly females [45]. These are fundamental factors driving the obesity epidemic [1]. Thus, exploring obesity experiences using representative sampling among undergraduate students will allow for the acquisition of information related to young Ghanaian adults. This knowledge will go a long way in informing strategies to combat the obesity epidemic and hopefully, related medical conditions among university students and the general young adult population.

We have mainly relied on the routine use of the body mass index (BMI) as an obesity measure. However, BMI has a limitation in differentiating between body composition and body fat distribution [46–49]. Alternative measures, including the bioelectrical impedance analysis (BIA) and BIA-derived body fat indices [50], like the body adiposity index [51] and relative fat mass (RFM) [52], have been proposed. These measures claim to adjust the limitations of BMI and alternatively represent cost-effective indices to appropriately identify individuals with accuracy close to that of underwater weighing [53] and dual-energy X-ray absorptiometry [54]. In particular, RFM and total percentage body fat (TPBF) have been validated as being a more accurate measure compared to BMI to estimate whole-body fat percentage, in addition to improving body fat-defined obesity misclassification among different population groups [55].

In this study, we have combined information contained in body mass index (BMI), TPBF and relative fat mass (RFM) to estimate an informative obesity prevalence. Because there is no single universally accepted measure of adiposity and each index has its drawbacks, we performed an evaluation analysis of waist-to-height derived RFM, corresponding to central fatness and TPBF corresponding to overall adiposity based on the routinely used weight-to-height derived BMI thresholds. By using data from the evaluation analysis assessing concordance and the estimate of measurement properties of TPBF and RFM with BMI, we combined this classification in a Bayesian framework. Thus, we reported an informative obesity prevalence corresponding to central and general adiposity, with much power. Our governing hypothesis was that a combined estimate of obesity in a Bayesian framework does not offer a more representative estimate than commonly used BMI, RFM and TPBF in isolation.

Because it is common in population surveys to have one or multiple measures investigating the same condition, the Bayesian framework has been useful in drawing inferences on disease prevalence and measurement properties while adjusting for the possibility of conditional dependence between several disease measures [56–58]. In practice, two aspects exist, that can be used to estimate uncertainty and improve the accuracy of population estimate of prevalence. The first is to use the prior information from existing studies, while the second requires the integration of multiple population-based measures into one estimate [57, 58]. In our case, we employed the second approach for this study.

## Methodology

This was a cross-sectional study undertaken at the Kwame Nkrumah University of Science and Technology (KNUST), Kumasi, between August 2018 and July 2019. All students provided written informed consent for their participation in the study. Ethical approval with reference ID (CHRPE/AP/030/19) was obtained from the Committee on Human Research, Publications and Ethics (CHRPE), School of Medicine and Dentistry, Kwame Nkrumah University of Science & Technology.

### Sample design

A two-stage stratified random sampling was used to select 1027 first to fourth-year undergraduate students aged 16–25 years. These students were selected to cover the six Colleges in KNUST including the College of Science (CoS), Art and Built Environment (CABE), Humanities and Social Sciences (CoHSS), Health Sciences (CoHS), Engineering (CoE), and Agric and Natural Resources (CANR). Students who were feverish, bodybuilders or highly trained athletes, and students with osteoporosis or oedema (swelling in the body) were excluded.

The targeted population consisted of undergraduate students’ population from 1st to 4th year of their academic level, across the six colleges of KNUST. The operational definition of a student’s year directly depended on the recruitment dates between January and April 2019. This period represents the second semester where first-year students have spent at least one complete semester in the college. We used a two-stage stratified cluster survey design. The study population and sampling consisted of the entire student population at KNUST (43,757) during the 2018/2019 academic year. Considering the low probability of sampling 5th and 6th -year students, available only for health sciences, were excluded from the sample. The first stage of clustering involved censoring all colleges with probabilities relative to the number of departments. From each selected college, a fixed number of departments was sampled. Eight students (two students, male and female, from levels 1st, 2nd, 3^rd,^ and 4th year) are sampled at random at the department level.

### Sample size

The required sample size to assess overweight/obesity prevalence among students was calculated assuming *p* = 0.18 [41], level of acceptable precision d = 0.05 (or ± 5%) at 95% CI corresponds to 227 which relate to 28 departments of 8 students in each. Using analysis of data from a previous study [41] within the same KNUST student population, the design effect was estimated at 1.9 for overweight/obesity. This was based on 6 clusters with an average of 50 students per cluster (n = 300) and an intra-cluster coefficient of 0.1. Considering these figures and while assuming a college response rate of 90% and individual students response rate at 85%, the actual sample size was estimated to be 83 departments of 8 students each (n = 664) with male and female students having equal proportion of being sampled. In the end, we recruited 1027 participants to increase the power of our estimate. Table 1 illustrates the minimum possible sampling expectations.

The equation for sample size calculation:

$$n=(z^2 \times p(1-p\left)\right(DEEF\left)\right)/\left(\right(j\left)\right(k\left)\right(l\left)\right(d^2\left)\right)$$ [59].

Z was taken at 1.96, j = is the expected response rate as a proportion (0.85 × 0.9); k is the average department size (n = 8). The proportion of the student population accounted for by the targeted of interest (l) was set at 0.8. DEFF is the design effect.

**Table 1:** Definition of sampling units and strata for the two-stage sampling procedure

Stage	Sampling Units (Colleges)	Strata	Minimum Required		Total existing units
			Units	students	
1	Sciences	1/9.4 *83 sampling fraction	8	64	11
	Art & Built Environment	1/9.4 *83 sampling fraction	8	64	10
	Humanities & Social Sciences	1.4/9.4 *83 sampling fraction	12	96	14
	Health Sciences	4/9.4 *83 sampling fraction	35	280	39
	Engineering	1/9.4 *83 sampling fraction	8	64	11
	Agric & Natural Resources	1/9.4 *83 sampling fraction	8	64	10
2	Sampling Units (Departments)	**Year/Level**	**Gender**		**Proportion**
		1st year	Male		0.50
			Female		0.50
		2nd year	Male		0.50
			Female		0.50
		3rd year	Male		0.50
			Female		0.50
		4th year	Male		0.50
			Female		0.50

### Data collection and anthropometric measurement

A structured questionnaire was used to collect data on socio-demographic characteristics, lifestyle risk factors and family history of obesity, diabetes and hypertension (Table 2). socio-demographic data included age, sex, year of study, and college. Lifestyle data included alcohol intake, smoking and exercise history. Height was measured with a portable height rod Stadiometer with students in a straight posture, feet placed together and flat on the ground. Waist circumference (WC) was measured using a tape measure at the point of the umbilicus and maximum gluteal protrusion. Each participant was asked to stand straight on the main unit of the OMRON BF511 Clinically Validated Full Body Composition Monitor with 8 high-precision sensors for hand-to-foot measurement (OMRON HEALTHCARE Co., Ltd.), looking straight, barefooted and with arms horizontally raised holding a display unit, extended at a 90° angle for weight, body fat mass (BFM) and TPBF estimation. The machine conforms to EN60601-1-2:2015 Electro Magnetic Compatibility (EMC) standard and uses the bioelectrical impedance, along with height, weight, age and gender information to generate results based on the OMRON’s data of body composition [60]. The Omron Full Body Sensor Body Composition Monitor and Scale estimates the TPBF by the Bioelectrical Impedance Method. The instrument sends an extremely weak electrical current of 50 kHz and less than 500 µA through the participant’s body to determine the amount of water in each tissue. The instrument takes measurements from both hands and feet to reduce the influence of water movement on body composition results. The output of the OMRON BF511 monitor included TPBF, relative visceral fat content, body mass index (BMI), and skeletal muscle. We included the TPBF and BMI output together with RFM for the analysis. RFM was calculated from WC and height:

$$\eqalign{RFM & =(64- (20*(height/waist\, circumference) \cr & + (12 \times ifelse (sex == female, 1, 0) }$$

Obesity was defined based on BMI thresholds for overweight (≥ 25 Kg/m^2^), and obesity (≥ 30 Kg/m^2^) according to the World Health Organization’s criteria [1].

### Statistical analyses

Patient characteristics were stratified by primary clusters (colleges). Counts and corresponding percentages were used to describe categorical variables and compared using the Chi-square test. Mean and standard deviations were used to describe continuous variables if they followed the Gaussian normal distribution. Median and interquartile ranges were used if otherwise distributed. Either the One-way analysis of variance or Kruskal-Willi’s test was used to compare continuous variables among primary clusters. Multiple comparison analysis with Bonferroni correction was performed when the probability value was < 0.05. Sex-stratified prevalence estimates for obesity were determined according to BMI thresholds and the corresponding TPBF and RFM thresholds for age. The Passing and Bablok regression analysis was used to evaluate the measurement agreement and possible systematic bias for TPBF and RFM against BMI [61]. The diagnostic accuracy of TPBF and RFM was estimated based on the optimal cut-off, sensitivity, and specificity analysis, considering the area under the curve (AUC) estimated with the receiver-operating characteristic curves (ROC) analysis. We integrated the results from TPBF and RFM for obesity definition based on BMI threshold in a Bayesian framework, to report a combined obesity prevalence. Twenty chains were used to sample 50,000 samples per chain (25,000 warmups and 25,000 post warmups). Posterior densities were estimated using the Hamiltonian Monte Carlo (HMC) method. Summaries of posterior distributions including the mean and 95% credible interval were used to interpret the results. Multiple logistic regression analysis was used to evaluate potential risk factors of overweight/obesity. A two-sided *p*-value of 0.05 was considered statistically significant. Statistical analyses were performed using R version 4.3.0 (2023-04-21 ucrt) and MedCalc software Bvba, version 18.9.1.

## Results

Table 2 displays the characteristics of the study participants by sampling strata. There was a significant over-representation of female students at CoHSS and underrepresentation at CABE and CoE (p-value < 0.001). The proportion of students that consume alcohol was comparatively low in CoHS (p-value = 0.039). Compared with CANR and CoHSS, a significant proportion of students from CoHS (31.2%), CoE and CoS (18.4% each) never engaged in regular exercise (p-value < 0.001). We observed significant variance in TPBF% measurements compared across the colleges (p-value < 0.001).

**Table 2:** Characteristics of the study participants by college

Variable	CABE (n = 118, 11.5%)	CANR (n = 81, 7.9%)	CoHSS (n = 150, 14.6%)	CoE (n = 114, 11.1%)	CoHS (n = 407, 39.6%)	CoS (n = 158, 15.4%)	p-value
**Gender**							
Female	44 (37.3)	46 (56.8)	105 (70.0)	47 (41.2)	243 (59.7)	89 (56.3)	**< 0.001**
Male	74 (62.1)	35 (43.2)	45 (30.0)	67 (58.8)	164 (40.3)	69 (43.7)	
Age (years)*	20.3 ± 2.0	19.9 ± 1.9	20.0 ± 1.9	20.0 ± 2.0	20.1 ± 1.9	19.7 ± 1.6	0.141
< 20 years	45 (38.1)	36 (44.4)	68 (45.3)	48 (42.1)	165 (40.5)	75 (47.5)	0.581
≥ 20 years	68 (57.6)	45 (55.6)	82 (54.7)	66 (57.9)	242 (58.5)	83 (52.5)	
**Year of Study**							0.459
1st	47 (39.8)	31 (38.3)	52 (34.7)	41 (36.0)	145 (35.6)	63 (39.9)	
2nd	22 (18.6)	18 (22.2)	29 (19.3)	25 (21.9)	105 (25.8)	28 (17.7)	
3rd	24 (20.3)	14 (17.3)	31 (20.7)	22 (19.3)	94 (23.1)	36 (22.8)	
4th	25 (21.2)	18 (22.2)	38 (25.3)	26 (22.8)	63 (15.1)	31 (19.6)	
Alcohol intake (yes)	11 (9.3)	8 (9.9)	12 (8.0)	9 (7.8)	13 (3.2)	11 (7.0)	**0.039**
Smoking (yes)	3 (2.5)	2 (2.5)	3 (2.0)	2 (1.8)	4 (1.0)	2 (1.3)	0.796
**Regular exercise**							
Never	16 (13.6)	8 (9.9)	11 (7.3)	21 (18.4)	127 (31.2)	29 (18.4)	**< 0.001**
Rarely	42 (35.6)	25 (30.9)	54 (36.0)	33 (28.9)	100 (24.6)	58 (36.7)	
Sometimes	46 (39.0)	34 (42.0)	62 (41.3)	42 (36.8)	138 (33.9)	57 (36.1)	
Almost everyday	14 (11.9)	14 (17.3)	23 (15.3)	18 (15.8)	42 (10.3)	14 (8.9)	
**Family history dx.**							
Hypertension	17 (14.4)	11 (13.3)	21 (14.0)	25 (21.9)	77 (18.9)	30 (19.0)	0.387
Diabetes	25 (21.2)	17 (21.0)	31 (20.7)	25 (21.9)	73 (17.9)	37 (23.4)	0.755
Obesity	12 (12.4)	7 (10.1)	14 (9.3)	11 (12.6)	26 (11.2)	12 (9.5)	0.933
**Measurements**							
Height (cm) *	170.2 ± 9.0	167.2 ± 7.5	164.9 ± 9.2	168.8 ± 9.5	165.6 ± 8.4	166.8 ± 8.7	**< 0.001**
Weight (Kg) *	65.3 ± 10.5	63.2 ± 8.9	63.9 ± 11.4	66.1 ± 11.2	63.2 ± 10.1	64.0 ± 10.4	0.110
WC (cm) *	73.9 ± 7.4	73.0 ± 5.9	73.0 ± 8.0	73.6 ± 8.2	72.6 ± 7.1	72.7 ± 6.9	0.499
BMI (Kg/m^2^) *	22.6 ± 3.3	22.7 ± 3.5	23.5 ± 3.7	23.2 ± 3.3	23.1 ± 3.6	23.0 ± 3.6	0.330
RFM (cm/cm) *	18.4 ± 4.5	19.0 ± 4.5	19.8 ± 4.5	18.5 ± 4.7	19.1 ± 4.8	18.8 ± 4.7	0.152
TPBF%^	22.0 ± 11.5	24.4 ± 11.1	28.2 ± 10.5	23.9 ± 10.3	25.8 ± 11.9	25.1 ± 11.9	**< 0.001**
Visceral fat ^	4.0 ± 2.1	3.7 ± 1.6	4.2 ± 1.7	4.4 ± 2.3	4.0 ± 1.9	4.0 ± 1.8	0.247

* Variables are presented as mean and standard deviations and compared using a one-way analysis of variance; ^ values are presented as means but compared using Kruskal-Willi’s test. All other variables are presented with count and corresponding proportions and compared using the Chi-square test. Multiple comparison analysis with Bonferroni correction was performed when the probability value was < 0.05

### Prevalence of being overweight and Obese based on standard BMI thresholds

Using BMI ≥ 25.0 Kg/m^2^, approximately 31% and 15% of female and male students were classified as overweight/obese. However, only 2.4% of male students and 8.0% of female students were classified as obese using BMI ≥ 30.0 Kg/m^2^ (Table 3). We observed a trend towards increased overweight/obesity with age. Among students < 20 years, 26.3% and 13.6% female and male, respectively, were classified as being overweight and/or obese. Among ≥ 20 years female and male students, 34.5% and 15.5% were classified as overweight and/or obese.

**Table 3:** Estimate of overweight/obesity prevalence based on body mass index

Obesity definition	Male students				Female students		
	< 20 years (n = 170)	≥ 20 years (n = 284)	Total (n = 454)	< 20 years (n = 266)		≥ 20 years (n = 307)	Total (n = 573)
**BMI criteria**							
25.0-29.9	20 (11.8)	36 (12.7)	56 (12.3)	54 (20.3)		77 (25.1)	131 (22.8)
≥ 30.0	3 (1.8)	8 (2.8)	11 (2.4)	16 (6.0)		29 (9.4)	46 (8.0)

The values shown are the actual number of individuals and gender-specific percentages

### Evaluation of RFM and BAI-derived TPBF based on BMI

**Fig. 1:**
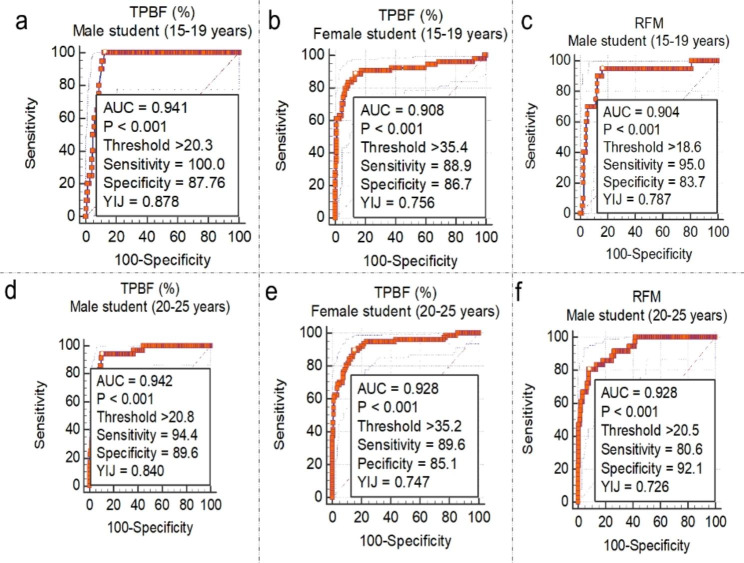
Threshold of TPBF and RFM estimates corresponding to WHO-defined BMI thresholds for overweight among male and female students

Passing and Bablok regression analysis are shown in Table S1. Here we emphasised the interpretation based on the observed random difference. The null assumption was that the observed random differences within ± 1.96 residual standard deviation (RSD) > 10%. Linearity between the variables was evaluated based on the custom test for linearity probability value (Table S1, Figure S1). Concordance was observed between TPBF and BMI (RSD = 1.60, ± 1.96 = -3.14 to 3.14) compared with RFM (RSD = 2.64, ± 1.96 = -5.17 to 5.17) among male students. Similarly, among female students, TPBF demonstrated good agreement with BMI (RSD = 2.23, ± 1.96 = -4.37 to 4.37) compared to RFM (RSD = 2.98, ± 1.96 = -5.83 to 5.83). The linearity test revealed a significant deviation from linearity between TPBF and BMI (p-value < 0.01) and between BMI and RFM (p-value > 0.05). The ROC curve analysis (Fig. 1) identified TPBF threshold values of > 20.3% and > 20.8% and was associated with high information values for defining being overweight (BMI > 29.9 Kg/m^2^) among male students: 15–19 years (AUC = 0.941, sensitivity = 100.0%, specificity = 87.8%) and 20–25 years (AUC = 0.942, sensitivity = 94.4%, specificity = 89.6%), respectively. Also, TPBF threshold values for defining obesity (BMI ≥ 30.0 Kg/m^2^) among 15–19 years and 20–25 years female students were > 35.4% and > 35.2%, respectively (Fig. 1).

**Fig. 2:**
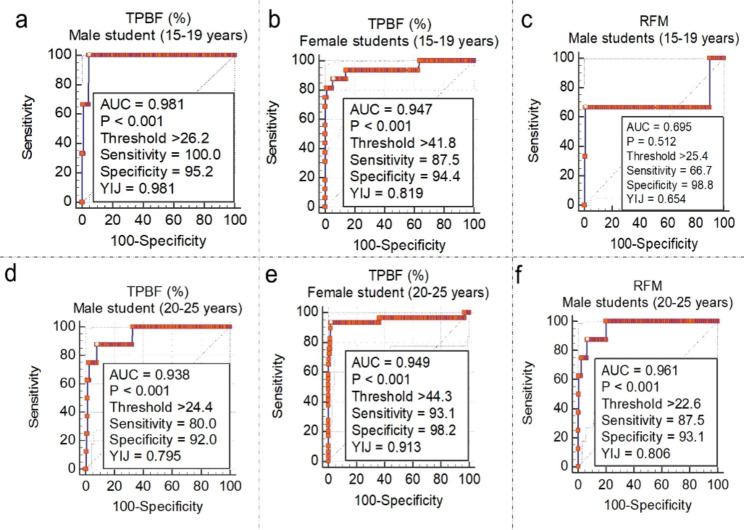
Threshold of TPBF and RFM estimates corresponding to WHO-defined BMI thresholds for obesity among male and female students

An optimal threshold value for TPBF > 26.2% (AUC = 0.981, sensitivity = 100.0%, specificity = 95.2%) for males (15–19 years) and > 24.4% (AUC = 0.938, sensitivity = 80.0%, specificity = 92.0%) had exceptional diagnostic accuracy for obesity (BMI ≥ 29.9 Kg/m^2^) among male students (Fig. 2). The TPBF threshold of > 35.4% and > 35.2 was optimal for defining overweight female students 15–19 years and 20–25 years, respectively. TPBF values > 41.8% and 44.3% were optimal for defining obesity among female students 15–19 years and 20–25 years.

**Fig. 3:**
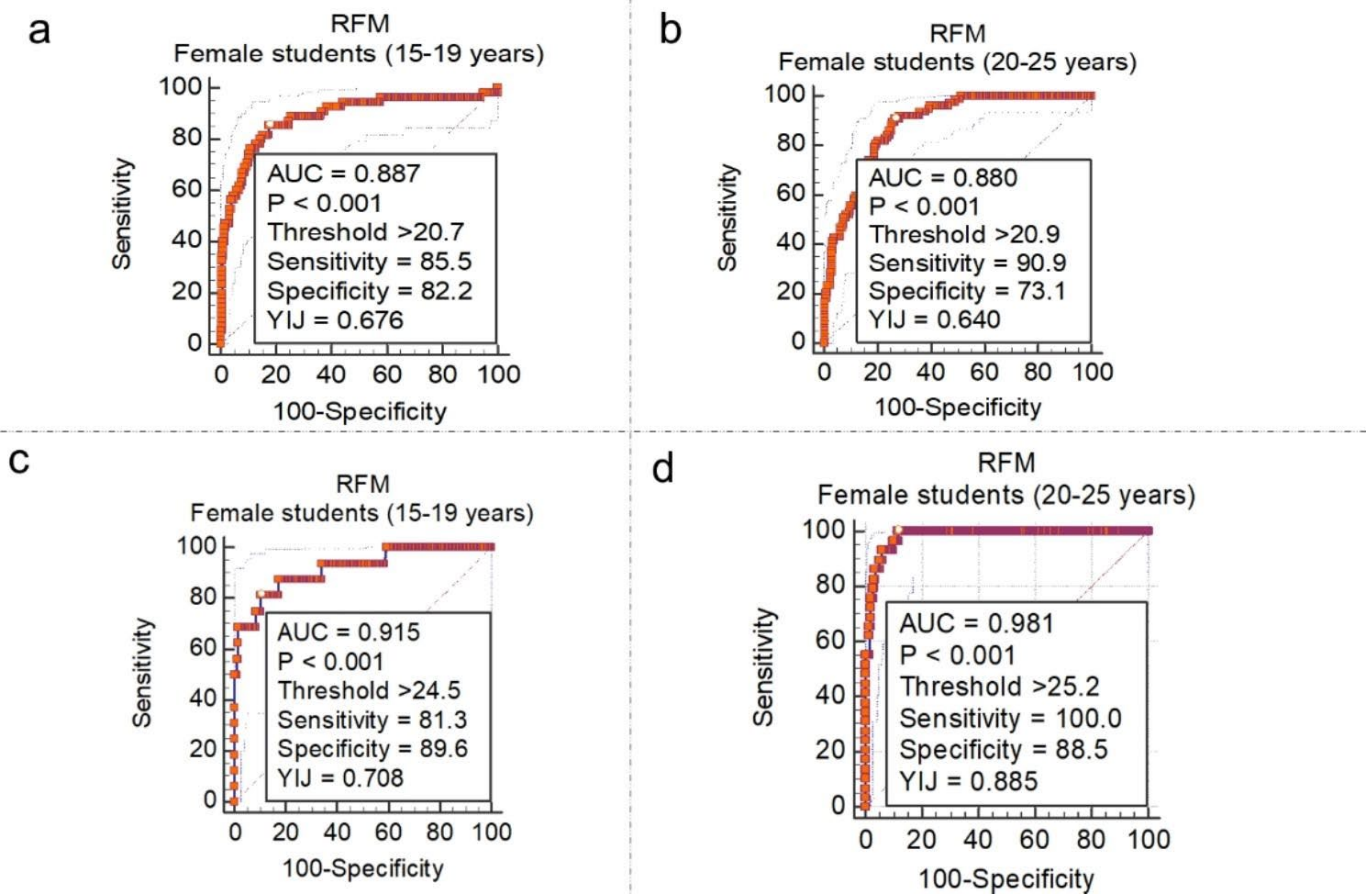
Threshold of RFM estimates corresponding to WHO-defined BMI thresholds for overweight and obesity among female students

The ROC curve analysis of RFM for defining overweightness revealed an optimal cut-off of > 18.6 and > 20.5 among 15–19 years and 20–25 years male students, respectively (Fig. 1c and f). RFM thresholds for defining obesity were > 25.4 and > 22.6 among male students 15–19 years and 20–25 years respectively (Fig. 2a and f). Among female students (Fig. 3a and b), RFM thresholds of > 20.7 and > 20.9 were associated with high information values for defining overweight among 15–19 years and 20 − 15 years, respectively. Moreover, obesity definition thresholds were > 24.5 and > 25.2, respectively among female students 15–19 years and 20 − 15 years (Fig. 3c and d).

**Fig. 4:**
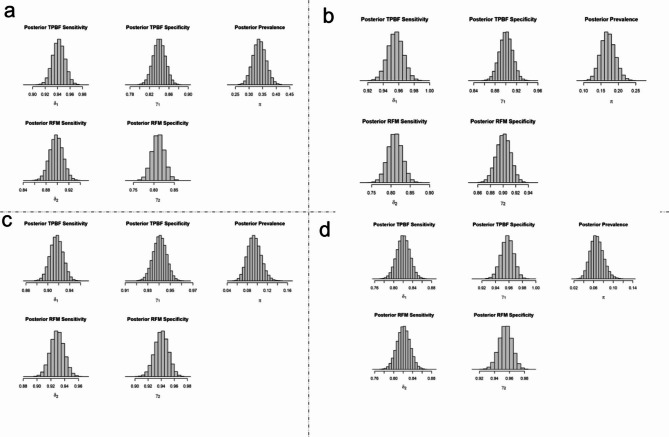
Marginal posterior density for the prevalence of obesity using combined data from TBPF and RFM. Note: π represents posterior prevalence using both TPBF and RFM data, δ1 represents sensitivity for TPBF data, γ1 represents specificity for TPBF data, δ2 represents sensitivity for RFM data, and γ2 represents specificity for RFM data

The concordance between BMI, TPBF and RFM for obesity classification was 84% (95% lower limit = 82.0%) among female students and 82.9% (95% lower limit = 80.5%) among male students. The findings of the posterior predictive checks using the simulated data are presented in Figure S3. The results of the Bayesian analysis suggest that the combined prevalence mean of overweight/obesity for TPBF and RFM were 33.8% (95%CI: 29.2-38.6%) among female students and 17.0% (95%CI: 13.1-21.3%) among male students. The combined prevalence mean of obesity for TPBF and RFM was 9.4% (95%CI: 6.9-12.2%) among female students and 6.7% (95%CI: 4.3-9.5%) among female students (Fig. 4).

### Factors associated with overweight/obesity among students

From the Multiple Logistic Regression analysis sex and family history of education were found to be consistent factors associated with general and central adiposity and percentage body fat distribution. The odds of being overweight were increased between 1.8 and 2.5, for women, depending on the classification criteria (Table 4). Family history of obesity was associated with increased odds of general obesity (OR = 3.48, 95%CI: 2.04–5.91), central obesity (OR = 1.98, 95%CI: 1.18–3.30) and high percentage body fat distribution (OR = 2.36, 95%CI: 1.42–3.94). Compared with first years students, the odds of central obesity and high percentage body fat were increased among third year students: OR = 2.77(1.59–4.82) and OR = 1.79(1.05–3.08), and fourth year students: OR = 3.26(1.76–6.04) and OR = 2.34(1.29–4.23), respectively.

**Table 4:** Multiple Logistic Regression analysis assessing factors associated with overweight/obesity prevalence among students

Factors	BMI Threshold		TPBF threshold		RFM threshold	
	OR (95%CI)	*p*-value	OR (95%CI)	*p*-value	OR (95%CI)	p-value
**Gender**						
Male	1		1		1	
Female	2.30(1.60–3.51)	**< 0.01**	1.78(1.26–2.51)	**0.001**	2.53(1.79–3.56)	**< 0.001**
**Age (years)**						
< 20 years	1		1		1	
≥ 20 years	1.41(0.84–2.36)	0.198	0.63(0.39–1.02)	0.060	0.78(0.49–1.24)	0.294
**Year of Study**						
1st	1		1		1	
2nd	0.62(0.35–1.09)	0.094	1.13(0.69–1.85)	0.625	0.95(0.59–1.53)	0.831
3rd	1.18(0.65–2.15)	0.577	2.77(1.59–4.82)	**< 0.01**	1.79(1.05–3.08)	**0.034**
4th	0.98(0.51–1.88)	0.944	3.26(1.76–6.04)	**< 0.01**	2.34(1.29–4.23)	**< 0.01**
**Regular exercise**						
Never	1		1		1	
Rarely	1.64(0.70–3.82)	0.702	1.01(0.49–2.11)	0.974	1.16(0.55–2.40)	0.692
Sometimes	1.29(0.64–2.63)	0.708	0.88(0.42–1.83)	0.735	1.13(0.54–2.30)	0.773
Almost everyday	0.91(0.35–2.40)	0.354	0.55(0.24–1.28)	0.166	0.78(0.34–1.78)	0.549
Alcohol intake (yes)	0.90(0.45–1.77)	0.750	0.94(0.91–1.76)	0.854	0.85(0.46–1.59)	0.613
Smoking (yes)	0.31(0.03–2.70)	0.286	0.20(0.03–1.75)	0.146	0.86(0.19–4.01)	0.852
**Family history dx.**						
Hypertension	0.78(0.48–1.28)	0.323	0.85(0.55–1.31)	0.458	0.69(0.44–1.07)	0.096
Diabetes	1.19(0.75–1.89)	0.457	1.22(0.80–1.86)	0.354	1.16(0.76–1.76)	0.496
Obesity	3.48(2.04–5.91)	**< 0.01**	2.36(1.42–3.94)	**0.001**	1.98(1.18–3.30)	**0.009**

OR- odds ratios, CI- confidence intervals, BMI- body mass index, TPBF- total percentage body fat, RFM- relative fat mass, dx – diseases

## Discussion

This study sought to investigate the prevalence and risk factors of obesity among undergraduate students using multiple adiposity indices in a Byersian framework. In general the prevalence of being overweight/obese in this age group of young adults was high: 33.8% among female students and 17.0% among male students. Significant association were found between being overweight/obese and potential factors including sex, family history of obesity and university level.

Using the combined informative estimate, we observed an obesity prevalence of 6.7% among male students and 9.4% among female students. More generally, 17.0% of male students and 33.8% of female students were classified as having weight status corresponding to abnormal central and general adiposity as well as high body fat accumulation. These estimates are within the obesity prevalence range of 1.7–19.0% as estimated by a previous study in the same population group using different anthropometric indices [41]. Among the university student population in Botswana [43] and Ghana [44], similar estimates of overweight and/or obesity prevalence have been reported. In a larger study representing university students from 22 countries [62], 14.1% and 5.2% of female students and 18.9% and 5.8% of male students were reported to be overweight and obese, respectively. These data highlight the significant burden of obesity among undergraduate university students, which has a potential future health impact. In line with the current 16.2%, tertiary enrolment rate in Ghana [63], the current estimate of obesity reflects a significant national obesity problem among the young adult population with significant future health consequences.

We believe our estimate may be a true reflection of the obesity burden among student populations. First, TPBF and RFM contain high information values for obesity and fat distribution classification. Second, RFM is less accurate than BMI in lean individuals [64] whiles BAI-derived TPBF is less accurate than BMI in obese individuals [65]. Thus, combining these measures in a population estimation of obesity would provide a value informed by a broader distribution of obesity and fat distribution among the population group. Third, we relied on prior information from the concordance between both RFM and TPBF with standard BMI thresholds and a previous study from the same population [41]. Finally, the method adapted for the estimation of the combined prevalence of obesity has been successfully applied elsewhere [66]. Because this approach could be more flexible and adaptable, there is a need to test its performance in other settings with other related adiposity estimates.

An important observation of concern was the proportion of overweight/obese female students, which was higher compared with male students. This observation is consistent in several other studies [40, 41, 43, 62], suggesting an increased risk for weight gain in young women and the critical need for interventions to prevent obesity and the host of associated adverse health outcomes. The evidence has been confirmed in several nationally representative surveys, where greater increases in weight are observed in young women aged 18 to 35 years compared with those seen in older women [67]. In a study among young Ghanain women aged between 15 and 24 years [20], overweight/obesity increased by 49% between 1993 and 2014 and projected a future prevalence of 35% by the year 2040. Fat deposition in women usually begins with the onset of puberty and continues unless consciously controlled [68]. Some studies has reported that female transition from adolescence to adulthood is associated with certain obesogenic dietary and physical activity behaviours to satisfy a historic valorization of large body size as a function of beauty, sexual attraction, prosperity, health and prestige [20, 69, 70].

In a prospective analysis of mother-daughter dyads and father-son dyads, the study reported a large and concerning increase in obesity rates over two generations of young adults, especially females [71]. These findings indicate that young adulthood represents periods of crucial importance regarding the establishment of life-long lifestyle habits and skills to control obesity. Studies have attributed this to a lack of knowledge and skills around food and nutrition, depression, anxiety, stress, satiety, neural responses, and possibly sleep patterns and premenstrual cravings [42–44, 67, 72]. Thus, there is the need to study these factors and their relationship with obesity among undergraduate students in Ghana, which can benefit future interventions.

We observed a trend in increasing obesity prevalence with academic level such that third- and fourth-year students had significantly increased obesity prevalence than first- and second-year students. Similar findings have been observed in other related studies [41, 73, 74] but not all [75]. This relationship may suggest the role of other factors of obesity associated with progressive academic level, which was not the focus of this study. We recommend future research to focus on changing lifestyle and eating habits of students related to progressive academic level. We replicated the association between obesity and family history and increased risk of obesity prevalence. This finding contributes to the evidence that genetics play an important role in the onset of obesity and the severity of obesity [76, 77]. In several studies, sendentary life has been strsongly associated with being overweight/obese [11, 17, 27, 28]. However, we did not find a significant association between being overweight/obese and students engagement in regular physical activity.

We would like to acknowledge some limitations of this study. First, Bayesian modelling is reliance on prior information, in our case we used the prior prevalence, concordance and diagnostic estimates obtained from the linked data and previous studies within the same population. As such, our analyses are limited by the accuracy of standard BMI thresholds for classifying obesity. Second, female students were over-represented in the dataset, which could bias the estimation of obesity prevalence. Although, we considered this in the analysis by reporting sex-specific prevalence estimates. Also, the generalizability of our estimate may be limited as data were obtained from only one tertiary institution in Ghana. This approach is significant to fill a gap in the current lack of consensus on the appropriate adiposity index and serves as the opportunity to unique data linkage and novel analytical techniques to improve obesity surveillance.

## Conclusion

As different adiposity indices become increasingly available, multiple indicators used in combination may offer a greater power to examine obesity prevalence. We have demonstrated this by integrating central adiposity and percentage body fat criteria relative to standard BMI thresholds in a Bayesian framework and reported high obesity prevalence, especially among female students. We also demonstrated that obesity prevalence increases with university level and among students with a family history of obesity. The study suggests that the prevalence of being overweight or obese is expected to increase in the coming years, leading to several health issues. It emphasizes the requirement for public health efforts and interventions at a national level to control the problem and its associated costs and co-morbidities. Furthermore, interventions against obesity should be customized to target the socio-demographic disparities highlighted in the study.

### Electronic supplementary material

Below is the link to the electronic supplementary material.

Supplementary Material 1

## Data Availability

The datasets and codes used and/or analysed during the current study are within the manuscript, and available at the GitHub repository (https://github.com/EvansKCCR/obesity_among_students).

## Abbreviations

RFM: Relative fat mass
TPBF: Total percentage of body fat
BMI: Body mass index
BIA: Bioelectrical impedance analysis
KNUST: Kwame Nkrumah University of Science and Technology
DEFF: Design effect
ROC: Receiver operative characteristics curve
AUC: Area under the curve
HMC: Hamiltonian Monte Carlo
RSD: Residual standard deviation
