# Supplementary material for: Prevalence and risk factors of obesity among undergraduate student population in Ghana: an evaluation study of body composition indices
Source: BMC Public Health. 2024 Mar 21;24:877. doi: 10.1186/s12889-023-17175-5 (PMC10958924; doi:10.1186/s12889-023-17175-5)
Supplement: Supplementary file 1 — Supplementary Material 1 [file 12889_2023_17175_MOESM1_ESM.docx]

**Prevalence and Risk factors of Obesity among Undergraduate Students Population in Ghana: An Evaluation Study on Body Composition Indices**

Christian Obirikorang^1,2*^, Evans Asamoah Adu^1,2^, Enoch Odame Anto^3,4^, Anthony Afum-Adjei Awuah^1,2^, Angela Nana Bosowah Fynn^2^, George Osei-Somuah^3^, Patience Nyarkoa Ansong^5^, Alexander Owusu Boakye^1,2^, Yaa Obirikorang^5^, Austin Gideon Adobasom-Anane^2^, Eric NY Nyarko^6^, Lois Balmer^4^

^1^Department of Molecular Medicine, School of Medical Science, Kwame Nkrumah University of Science and Technology (KNUST), Kumasi, Ghana

^2^Kumasi Centre for Collaborative Research, Kumasi, Ghana

^3^Department of Medical Diagnostics, Faculty of Allied Health Sciences, Kwame Nkrumah University of Science and Technology, Kumasi, Ghana

^4^ Centre for Precision Health, School of Medical and Health Sciences, Edith Cowan University, Western Australia, Australia

^5^Department of Nursing, Faculty of Health Sciences, Garden City University College, Kumasi, Ghana

^6^Department of Chemical Pathology, University of Ghana Medical School, University of Ghana

***Corresponding Author**

Christian Obirikorang

Department of Molecular Medicine

School of Medical Science

Kwame Nkrumah University of Science and Technology (KNUST)

Kumasi, Ghana

[krisobiri@gmail.com](mailto:krisobiri@gmail.com)

**Supplementary Data**

**Table S1 Results from Passing and Bablok regression analysis comparing BMI and RFM to BIA-derived TPBP.**

| **Analysis Output** | **BMI vs TPBF** | **RFM vs TPBF** | **RFM vs BMI** |
| --- | --- | --- | --- |
| **Male Students** | | | |
| Regression equation: | y = -42.93 + 2.64x | y = -15.16 + 1.79x | y = 11.22 + 0.64 x |
| Intercept (95%CI) | -42.93 (-47.74 to -39.67) | -15.16 (-18.17 to -13.20) | 11.22 (10.14 to 11.90) |
| Slope (95%CI) | 2.64 (2.50 to 2.87) | 1.79 (1.66-1.95) | 0.64 (0.60 to 0.71) |
| RSD (±1.96 RSD) | 1.60 (-3.14 to 3.14) | 2.64 (-5.17-5.17) | 1.53 (-2.99 60 2.99) |
| p-value (CTL) | 0.19 | 0.04 | 0.01 |
| Rho (95% CI) ^a^: | 0.799 (0.76 to 0.83) | 0.649 (0.59 to 0.70) | 0.74 (0.69-0.78) |
| **Female Students** | | | |
| Regression equation | y = -17.75 + 2.18x | y = -1.43 + 1.69 x | y = 8.15 + 0.75x |
| Intercept (95%CI) | -17.75 (-20.18 to -15.03) | -1.43 (-3.72 to 0.90) | 8.15 (7.21 to 9.11) |
| Slope (95%CI) | 2.18 (2.06-2.23) | 1.69 (1.57 to 1.81) | 0.75 (0.70 to 0.80) |
| RSD (±1.96 RSD) | 2.23 (-4.37 to 4.37) | 2.98 (-5.83 to 5.83) | 1.93 (-3.78 to 3.78) |
| p-value (CTL) | <0.001 | 0.420 | <0.01 |
| Rho (95% CI): | 0.85 (0.82-0.87) | 0.73 (0.69 to 0.77) | 0.79 (0.76 to 0.821) |

***Intercept=measure of systematic differences; slope= measure of proportional differences; residuals = random differences. RSD = residual standard deviation; CTL= custom test for linearity.***


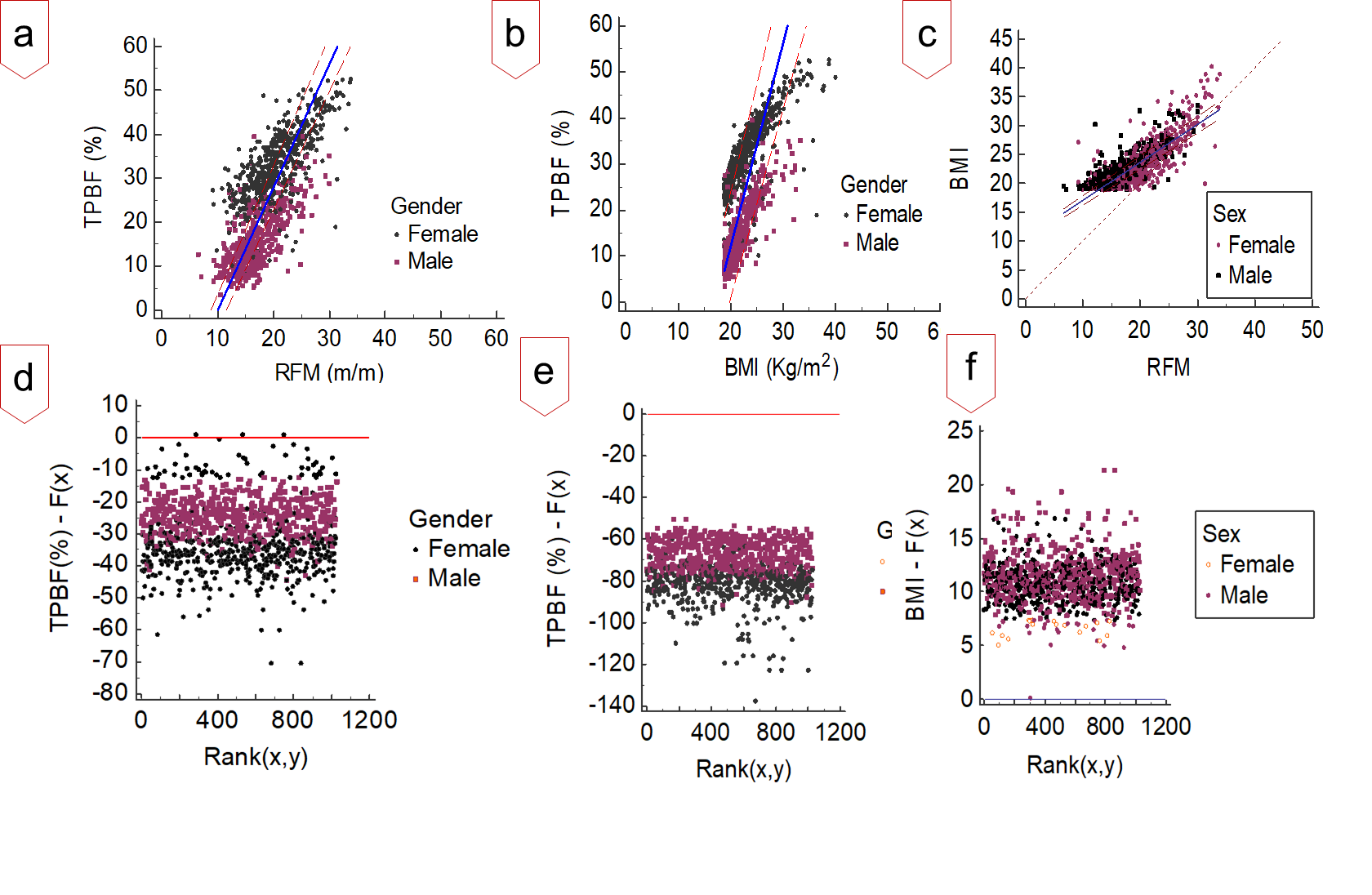


**Figure S1: Scatter plot with regression line and confidence band of the regression line (a and b) showing the association between TPBF with BMI and RFM. Residual plot (c and d) represents distribution of differences around fitted regression line.**

**
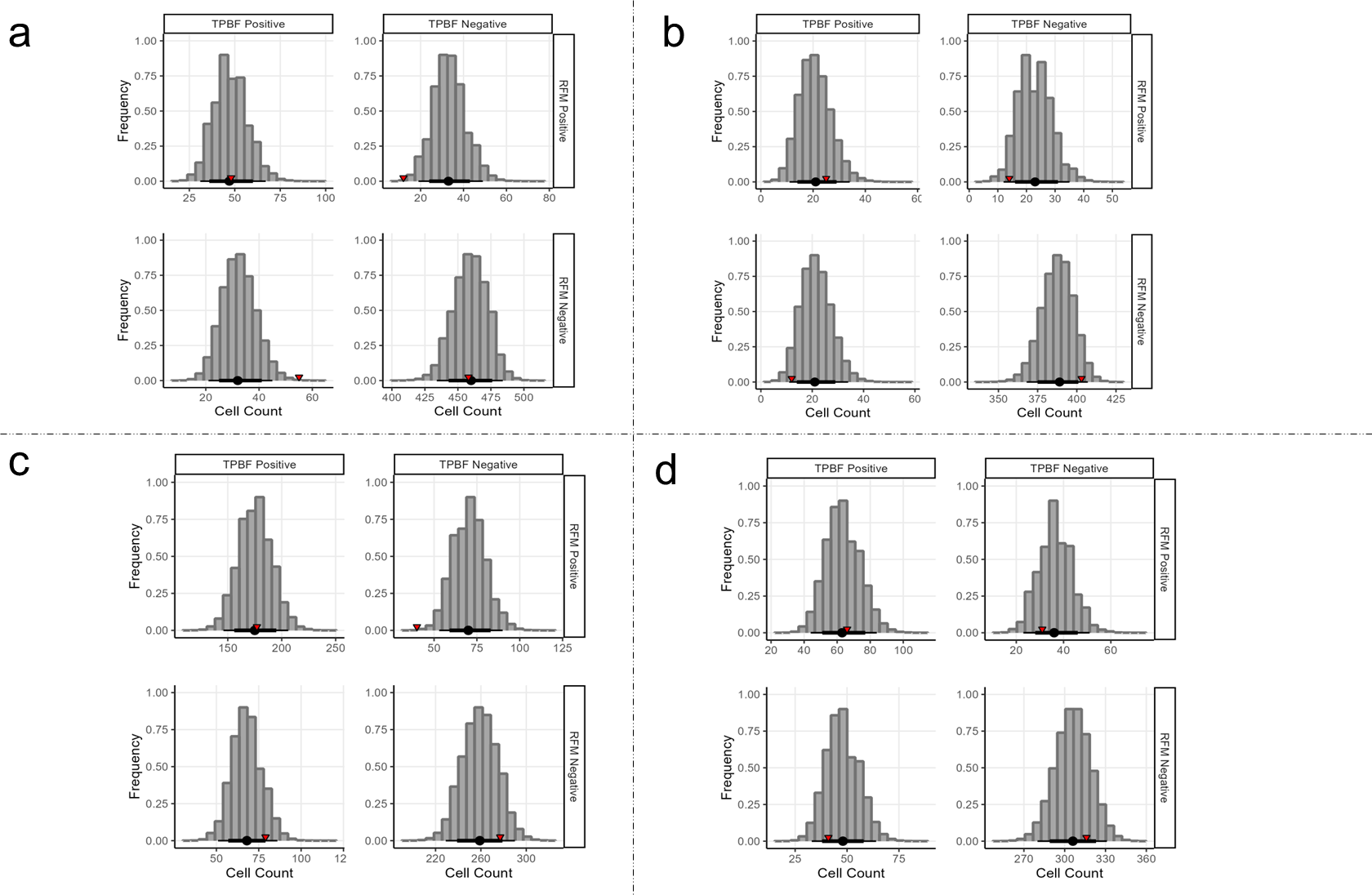
**

**Figure S2 Predictive probability checks between TPBF and RFM threshold for obesity definition corresponding to standard BMI threshold ≥25.0 Kg/m^2^ and ≥30.0 Kg/m^2^ among male and female students. a) concordance between TPBF and RFM for classification of obesity among female students; b) concordance between TPBF and RFM for classification of obesity among male students; c) concordance between TPBF and RFM for classification of overweight among female students; d) concordance between TPBF and RFM for classification of obesity among female students.**
